# Reducing the Individual Carbon Impact of Video Streaming: A Seven-Week Intervention Using Information, Goal Setting, and Feedback

**DOI:** 10.1007/s10603-023-09536-9

**Published:** 2023-02-13

**Authors:** B. T. Seger, J. Burkhardt, F. Straub, S. Scherz, G. Nieding

**Affiliations:** Developmental Psychology Department, University of Würzburg, Röntgenring 10, 97070 Würzburg, Germany

**Keywords:** Behaviour change, Video streaming, Climate communication, Goal setting, Feedback

## Abstract

This online intervention study examined whether system- and action-related information alone, together with goal setting, or together with goal setting and feedback helps people change their video streaming activities in a pro-environmental way. The participants (*N* = 92) documented their video streaming activities for one week prior to the intervention (week 1), three weeks after the onset of the intervention (weeks 2–4), and in a follow-up period two weeks later (week 7). A reduction of greenhouse gas emissions associated with video streaming was observed over the course of the intervention, together with reduced streaming durations and lowered resolution settings across all groups. There were no differences between the groups. It appears that as regards video streaming, information combined with self-monitoring has considerable potential to change individual behaviour and its associated ecological impact.

## Pathways to Climate-Friendly Video Streaming

Within the past decade, particularly during the ongoing Covid-19 pandemic, Internet use in general and video streaming in particular have dynamically increased (e.g., Lemenager et al., 2021). Among German residents aged 14 and above, for example, 72% have indicated using the Internet and 30% have indicated streaming video content every day in 2020 (Beisch & Schäfer, 2020). This resulted in an average 55 streaming minutes per person and day, compared to 42 streaming minutes in 2019. Two-thirds of the interviewed adolescents and young adults (aged 14 to 29) indicated streaming video content on a daily basis, associated with an average 130 streaming minutes per person and day in 2020 (Beisch & Schäfer, 2020). In line with Suski et al. (2020), we defined video streaming as watching videos from online sources, including media libraries, advertised video-on-demand (VoD) platforms (e.g., YouTube), subscription-VoD platforms (e.g., Netflix), and social media platforms (e.g., Facebook). As videos are among the most data-intensive types of web content, their share of global energy use and associated greenhouse gas (GHG) emissions is becoming increasingly relevant to global warming. According to Shehabi et al. (2014), one hour of video streaming leads to an average emission of 420 grams of carbon dioxide equivalents (CO_2_e). In 2018, video streaming activities accounted for 60% of worldwide data traffic and caused 306 megatons of CO_2_e, which was comparable to the annual emissions of Spain (Efoui-Hess, 2019).

As a consequence, providers, users, and policy makers should participate in efforts to reduce the GHG emissions associated with video streaming (Efoui-Hess, 2019; Preist et al., 2019; Suski et al., 2020). In line with Akenji’s (2014) attitude-facilitator-infrastructure framework of sustainable consumption, three different approaches should be followed here. First, the infrastructure needs to be appropriate to enable climate-friendly video streaming. This includes energy-efficient datacentres that run on renewable sources; providers are accountable at this point, while governments must put suitable framework conditions in place. In a broader sense, the supply of end-user devices also needs to be addressed, one of the major challenges being the reduction of the material footprint associated with the life cycle of devices (Suski et al., 2020). Here, producers need to take responsibility, and political institutions should push this by supporting reparation and recycling of devices through the legal system, for instance. Second, sustainable behaviour should be facilitated. Platform providers have various design options here, including climate-friendly default settings for provided videos or an audio-only option for music videos or podcasts (Preist et al., 2019). Policy makers can support such business practices by eco-labels or financial incentives.

Third, stakeholders—including but not limited to individual users—need to be ready to adopt climate-friendly practices in the context of video streaming, especially when the infrastructure is insufficient and the facilitators are weak (Akenji, 2014). Individual users may either follow a *sufficiency* strategy by reducing their streaming duration, or increase the *efficiency* of video streaming by reducing the carbon impact per hour. Suski et al. (2020) identified two leverage points for efficiency at the user level: the device used for streaming and the chosen video resolution. In general, smaller screens and lower resolutions are associated with a lower carbon impact.

In the present experiment, we investigated whether informational strategies of behaviour change (Abrahamse & Matthies, 2013; Nemati & Penn, 2020) can reduce the impact of individual video streaming by fostering efficiency- and sufficiency-related user behaviours. Such strategies include (but are not limited to) information provision, goal setting, and feedback. In the following, we outline how an intervention composed of these three strategies can support changes to individual video streaming behaviour.

### Information Provision

Logically, being aware of the environmental impact of video streaming requires having knowledge of these problems, and having relevant information should increase such knowledge. From an empirical perspective, there is meta-analytic evidence that information provision is positively associated with pro-environmental behaviours (Hines et al., 1987; Osbaldiston & Schott, 2012). However, conceptual work on this issue (e.g., Abrahamse et al., 2005; Kollmuss & Agyeman, 2002; Lehman & Geller, 2004) suggests that the causal relationship between information provision, problem awareness, and pro-environmental behaviour change is not as trivial as it may seem. On the one hand, environmental knowledge is behaviour-distal (Geiger et al., 2019), which implies that other variables, including trait variables such as value orientations, have a moderating effect on the relationship between information, knowledge, and behaviour (e.g., Bolderdijk et al., 2013). On the other hand, knowledge is not a unitary construct. Frick et al. (2004) have discerned three different types of environmental knowledge: System knowledge refers to ecosystems and ecological problems caused by human behaviour; action knowledge points up behavioural options that help solve or mitigate these ecological problems; and effectiveness knowledge comprises information on the ecological impact of each behavioural option. To be effective, an information intervention should address at least system and action knowledge. For example, Dogan et al. (2014) found that an intervention that includes system-, action-, and effectiveness-related information increases the intention to implement ecologically sufficient driving behaviours (e.g., driving below the speed limit on highways) more than mere recommendations to adopt these behaviours (i.e., providing action-related information only).

According to Lehman and Geller (2004), informational interventions should be tailored to a specific behaviour in a specific situation of the person affected. Stage models of behaviour change (Bamberg, 2013; Pelletier & Sharp, 2008) provide ideas on how such tailoring can be implemented to carry out behaviour changes in the long run. Given that the public probably has limited knowledge of the ecological consequences of video streaming (Arend & Buchholz, 2021; Suski et al., 2020), information should be tailored to early stages of behaviour change such as the *detection phase* in Pelletier and Sharp’s (2008) model. Accordingly, system-related information (e.g., how video streaming is associated with global warming) should be provided to increase people’s problem awareness. The next stage, referred to as the *decision phase*, is characterised by a shift from problem- to solution-oriented considerations. Here, action-related information (e.g., that one can reduce the impact of video streaming on global warming by using other devices or changing the resolution settings) is particularly helpful. Bamberg’s (2013) model of self-regulated behaviour change provides a *predecisional stage* with the perceived negative consequences of the current behaviour and the perceived feasibility of a goal-directed behaviour change as crucial predictors. Tailored interventions for this phase should hence include system-related information to increase people’s awareness of the climatic consequences of video streaming and action-related information to increase the perceived feasibility of behaviour changes that mitigate these consequences.

### Goal Setting and Feedback

Goal setting is a common approach to foster pro-environmental behaviour and energy conservation in particular (e.g., Becker, 1978; Brandsma & Blasch, 2019; Harding & Hsiaw, 2014; Loock et al., 2013). Goal-setting theory (Locke & Latham, 1990, 2019) predicts that envisaged behaviour changes are most pronounced when goals are specified for a certain behaviour in a certain context and are ambitious yet attainable. Feedback has been identified as a crucial moderator of goal-directed performance because it enables people to track their progress and adjust their goals if needed (Kluger & DeNisi, 1996; Locke & Latham, 2019). In line with this, the combination of goal setting and feedback is frequently encountered in the literature on interventions for energy conservation (Becker, 1978; Brandsma & Blasch, 2019; Karp et al., 2016; McCalley & Midden, 2011; van Houwelingen & van Raaij, 1989). A meta-analysis of feedback interventions for energy conservation (Karlin et al., 2015) yields a small positive effect in total, with most (but not all) included studies reporting less energy use after feedback. This effect is larger when such feedback is combined with goal setting and when the feedback is itself goal-based by comparing the actual energy conservation or GHG reduction with that envisaged.

Goal setting also links to theoretical approaches to behaviour change. According to the stage model of self-regulated behaviour change (Bamberg, 2013), people form a goal intention (e.g., “I plan to reduce the energy consumption of my video streaming activities by 20%”) that enables them to proceed from the predecisional stage to the second one, referred to as the *preactional stage*. Hence, goal setting can be a useful strategy at this point. In the next step, people choose behavioural strategies (by using their action knowledge, for instance) and form a behavioural intention, which marks the entrance to the *actional stage*. This third stage is characterised by the implementation of specific behaviours in specific situations, resulting in *implementation intentions* (Gollwitzer, 1999) that directly precede the envisaged behaviour (e.g., “When watching YouTube tonight, I am going to switch off the Autoplay function and lower the resolution setting from 720 to 480 pixels”). Likewise, in Pelletier and Sharp’s (2008) model of motivated behaviour change, a goal intention marks the transition from the decision phase to the *implementation phase*; these authors also recommend both goal setting and implementation intentions as adequate intervention strategies. Pelletier and Sharp (2008) argue further that combining goal intentions with implementation intentions may be sufficient for a long-term behaviour change if the person is motivated; feedback would not be necessary in that case. By contrast, Bamberg’s (2013) model provides a fourth, *postactional stage* where people may face challenges to maintain the implemented behaviour change and recover from relapses. The success of both maintenance and recovery depends on people’s *self-efficacy* (Schwarzer, 2008), referring to the extent to which they believe in their ability to meet these behavioural challenges (Bandura, 1997). An increased self-efficacy is associated with feedback in general (Kluger & DeNisi, 1996) and with the combination of goal setting and feedback in particular (Locke & Latham, 2019).

### Combining Information Provision with Goal Setting and Feedback

For the present study, we expect that a tripartite intervention composed of information, goal setting, and goal-based feedback helps save energy and reduce GHG emissions in the context of video streaming. Regarding pro-environmental behaviour in general, there is empirical support for this bundle of interventions: Osbaldiston and Schott’s (2012) meta-analysis yields higher effect sizes for information-goal setting and information-feedback combinations than for goal setting and feedback alone. Abrahamse et al. (2007) designed such a tripartite intervention to help households reduce their overall energy consumption associated with oil, gas, and electricity. Compared to a no-intervention control group, households in the intervention group reported significantly lower energy use five months after having received problem-related information and tailored action-related information, a goal to reduce their energy use by 5%, and interim feedback after two months.

We apply this intervention bundle to the specific context of video streaming, which is rather novel in the area of promoting pro-environmental behaviour (Suski et al., 2020). Unlike Abrahamse et al. (2007), we employ a graded study design by comparing the tripartite intervention to a bipartite intervention that combines information with goal setting, and a baseline intervention where only information is provided. The information intervention addresses system and action knowledge (Frick et al., 2004). The goal and feedback used in our intervention are expressed in GHG emissions (grams CO_2_e) associated with video streaming. We decided to employ a 20% GHG reduction goal, which equals the more ambitious electricity-saving goal in Becker’s (1978) classical experiment. As part of the goal setting intervention, implementation intentions (Gollwitzer, 1999) were used to support the implementation of goal-directed behaviour change (Bamberg, 2013; Pelletier & Sharp, 2008). Given that public awareness of the climate impact of video streaming is rather low, we expect a significant reduction of GHG emissions in all experimental conditions (Hypothesis 1). We further hypothesize that goal setting adds to the beneficial effect of information (Hypothesis 2) and that feedback adds to the beneficial effect of goal setting and information (Hypothesis 3). In addition, we conduct two exploratory analyses. First, we want to know whether feedback affects goal attainment. Second, we explore the extent to which users apply efficiency- and sufficiency-related behaviours by analysing the effects of the graded interventions on streaming duration, device choice, and resolution setting.

## Method

### Sample

We conducted an a priori power analysis using G*Power (Version 3.1.9.2; Faul et al., 2007). As a heuristic for the effect size, we used the reduction goal of 20% as the mean difference and a standard deviation of 50% of total GHG emissions, which approximate the values obtained in Loock et al.’s (2013) large sample. The effect size to be obtained is δ = 0.4 (*f* = 0.2). For a mixed 3*3 design, achieving this effect size with a 95% power would require at least 66 participants (22 per condition) for a main effect within participants and 81 participants (27 per condition) for a within-between interaction.

The initial sample consisted of 106 participants, with *N* = 92 (86.8%) completing our seven-week online experiment (week 1: baseline, weeks 2–4: intervention, week 7: follow-up after a two-week break). Thirty-one participants were in the information-only (i1) group, 29 were in the information plus goal setting (i2) group, and 32 were in the information plus goal setting plus feedback (i3) group, implying that each group reached the minimum size of 27 specified in the power analysis. The participants were between 18 and 57 years old, the majority being young adults (mean age = 22.93, *SD* = 5.59). Females were overrepresented (63%) in this sample. The participants were acquired online via the psychological research system of the University of Würzburg (2021). The recruitment text disclosed that video streaming behaviour was addressed; however, we avoided any information referring to the pro-environmental objective of the study. All participants were native German speakers or spoke German at a comparable level. Forty-eight participants (52.2%) were undergraduate psychology students who received partial course credit for participating; the remaining participants received a €30 compensation payment. Data collection took place between January and March 2021 (*n* = 32) and between June and August 2021 (*n* = 60). Age, gender, proportion of psychology undergraduates, and data collection wave were approximately equally distributed across groups (see Table 1). All participants provided informed consent prior to the experiment.

**Table 1:** Sample Characteristics

			Total sample	Information only (i1)	Information + Goal setting (i2)	Information + Goal setting + Feedback (i3)
	*N* (%)		92 (100%)	31 (33.7%)	29 (31.5%)	32 (34.8%)
Age (years)		Mean *(SD)*	22.93 (5.59)	24.32 (8.69)	22.41 (3.48)	22.06 (2.18)
Frequency of tracking tool use^a^		Mean *(SD)*	2.84 (1.74)	2.87 (1.78)	2.93 (1.73)	2.72 (1.75)
Gender	Female	*n* (%)	58 (63.0%)	20 (64.5%)	21 (72.4%)	17 (53.1%)
	Male	*n* (%)	32 (34.8%)	10 (32.3%)	8 (27.6%)	14 (43.8%)
	Other/not specified	*n* (%)	2 (2.2%)	1 (3.2%)	0 (0%)	1 (3.1%)
Compensation mode	Psychology course credit	*n* (%)	48 (52.2%)	14 (45.2%)	18 (62.6%)	16 (50%)
	Compensation payment	*n* (%)	44 (47.8%)	17 (54.8%)	11 (37.4%)	16 (50%)
Data collection wave	January–March 2021	*n* (%)	32 (34.8%)	10 (32.3%)	10 (34.5%)	12 (37.5%)
	June–August 2021	*n* (%)	60 (65.2%)	21 (67.7%)	19 (65.5%)	20 (62.5%)

^a^1 = never, 2 = rarely, 3 = occasionally, 4 = often, 5 = always

### Measurement

We used a diary method implemented in SoSciSurvey (Leiner, 2019) to assess video streaming. The participants completed one survey page per day where they indicated the streaming duration and specified the resolution setting for each of four devices (smartphone, tablet, laptop/PC, and smart TV) and three platform types (advertised VoD, subscription-VoD, and social media and private websites). To indicate the resolution, participants chose from a dropdown menu that contained exact frame rate values (pixel) and the verbal categories “low,” “medium,” “high,” and “ultra HD” used by Netflix, in particular. Participants were offered a manual that provided information on how to view and change resolution settings (based on Köhn et al., 2020). They were also invited (but not obliged) to use a tracking application that indicated their streaming duration (ActionDash, MindTheTime, or TinyStopwatch, depending on the operating system). Using a 5-point Likert scale, we asked how often the participants used such an application; there was no difference between the experimental groups in this regard (see Table 1). In line with Suski et al. (2020), we instructed participants to divide the streaming duration by the number of co-viewers when watching a video together on the same screen. We also asked the participants to indicate only their streaming activities during leisure time, excluding study- or work-related activities such as online lectures or virtual meetings.

To calculate the GHG emissions associated with each participant’s video streaming activities in one week, we multiplied streaming hours by the electricity use of data traffic and device and the GHG emissions per electricity unit, then added up the resulting GHG values per device, platform type, and day (Table 2). For the electricity use per device, we referred to the values provided by Suski et al. (2020). For resolution settings described verbally, we estimated the data traffic based on Netflix (2021) for subscription-VoD platforms and Hindy (2019) and Ilumba (2020) for the other two platform types. Finally, electricity use was translated into GHG emissions using the value of 447 grams CO_2_e per kilowatt hour, as determined by Moro and Lonza (2018).

**Table 2:** Calculation of greenhouse gas emissions of streaming activities per participant and week

$${E}_{A,B}=\sum_{i=1}^{4}\sum_{j=1}^{7}\sum_{k=1}^{3}{t}_{i,j,k}({P}_{i}+Q*R)\gamma$$		
Variable	Unit	Legend
${E}_{A,B}$	Grams of carbon dioxide equivalents	Greenhouse gas emission associated with the video streaming activities of participant A in week B
${t}_{i,j,k}$	Hour	Streaming duration for end user device *i* on day *j* on platform type *k*
${P}_{i}$	Kilowatt	Electricity use of device *i*
$Q$	Gigabyte per hour	Data traffic, depending on resolution setting
$R$	Kilowatt hour per gigabyte	Electricity use associated with data traffic
$\gamma$	Grams of carbon dioxide equivalents per kilowatt hour	Greenhouse gas emission associated with electricity use in the European Union (Moro & Lonza, 2018)

We are aware that the diary method can be seen as an intervention on its own, involving the participants’ self-monitoring and self-feedback of their streaming duration and resolution settings in all conditions. Therefore, the interpretation of our results in terms of experimental conditions is limited. Moreover, we neglected two sources of GHG emissions in this study, namely those caused by the providers’ datacentres and those associated with the production and transport of end-user devices. At these points, individual users can reduce GHG emissions by purchasing refurbished devices, using devices for longer or repairing them, or subscribing to more climate-friendly streaming platforms. However, our focus was not on such acquisition behaviours, but rather on the ongoing use of devices and platforms.

### Intervention

Information provision was performed in two steps. The participants watched a video (The Shift Project, 2019, https://www.youtube.com/watch?v=JJn6pja_l8s; 2′30 min duration) that focuses on the ecological consequences of Internet use in general and video streaming in particular (i.e., it provides system knowledge). Then, we provided action knowledge using a text page that contained six tips to reduce GHG emissions: using the laptop or smartphone instead of the smart TV when watching as a side-line; reducing the resolution; deactivating the AutoPlay function; not streaming on more than one device at the same time; streaming music on audio instead of video platforms; and watching videos together on the same screen.

At the end of week 1, participants who received a goal-setting intervention were asked to reduce their GHG emissions associated with video streaming by 20% compared to the baseline. In addition, they were invited to note three situation-specific measures that would help them achieve this goal (implementation intentions; Gollwitzer, 1999). Feedback was implemented at the end of each week and included the total carbon impact expressed in grams CO_2_e. To illustrate the carbon impact, we translated it into car-driving kilometres based on data provided by the Federal Environmental Agency of Germany (Umweltbundesamt, 2021; 154 grams CO_2_e per kilometre) and full loadings of a smartphone battery (Hanna et al., 2019; 8.4 grams CO_2_e per loading). The feedback also specified how the carbon impact had changed compared to baseline and whether or not the participant had reached the 20% reduction goal.

### Design and Procedure

This study comprised three phases: a baseline measurement phase (week 1), an intervention-plus-measurement phase (weeks 2–4) and, after a two-week break, a follow-up measurement phase (week 7). The participants were randomly assigned to one of three intervention groups. The i1 group only received information, the i2 group received information and goal setting, and the i3 group received information, goal setting, and feedback. The information and goal-setting interventions were provided at the end of week 1. Baseline feedback (i.e., the total GHG emissions of week 1) was provided to both the i2 and i3 groups so that these two groups had a quantifiable reduction goal. They were also reminded of their 20% reduction goal and their individual implementation intentions after weeks 2, 3, and 4. The i3 group also received the goal-related feedback at these times. Sociodemographic data of all participants were gathered at the end of week 7, after which they were given a complete explanation of the study and final feedback on their GHG emissions over the entire course of the study.

Prior to the experiment, we distributed an initial questionnaire via SoSciSurvey in which participants were informed about the goal of the study, gave consent, received a participant code to preserve anonymity, and subscribed to a mailing list. The subscribers then received regular e-mails on Tuesday, Thursday, and Sunday of each measurement week with the link to the diary pages. On particular dates, they also received the main intervention (Sunday week 1), goal reminder (Sundays weeks 2–4, i2 and i3 groups only), interim feedbacks (Sundays weeks 2–4, i3 group only), and final questionnaire and feedback (Sunday week 7). Participants whose diary entries were missing at the end of a measurement week received an extra e-mail reminding them to enter the missing data.

## Results

The survey data have been published in an open repository (https://osf.io/kazy7/). For all dependent variables other than goal attainment, we conducted a mixed analysis of variance (ANOVA) with experimental group (i1 vs. i2 vs. i3) as the between- and time as the within-participant factor. For the time factor, we used the week 1 means as baseline (t1), the averaged means of weeks 2–4 as intervention (t2), and the week 7 mean as follow-up (t3). We decided to average the available means per person for weeks 2–4 because we received incomplete data from six participants over these three weeks, which would have resulted in reduced power when analysing each week separately. Using only the week 4 values for t2 did not yield significantly different results in terms of GHG emissions. The mean carbon impact of all participants at t1 was 1897 grams CO_2_e (*SD* = 1,366); there were no significant differences between the groups, *F*(2, 89) = 1.478, *p* = 0.23. The results are summarized in Table 3.

**Table 3:** Greenhouse gas emissions, video streaming duration, and resolution settings per experimental group and measurement time (*N* = 92)

	Baseline (t1)	Intervention (t2)	Follow-up (t3)
Mean (*SD*) weekly greenhouse gas emissions in grams of carbon dioxide equivalents			
Total	1897 *(1366)*	1187 *(913)*	1274 *(1112)*
Group i1^a^	2211 *(1288)*	1644 *(1086)*	1715 *(1225)*
Group i2^b^	1863 *(1509)*	1000 *(742)*	1202 *(1114)*
Group i3^c^	1625 *(1279)*	914 *(702)*	913 *(850)*
Mean (*SD*) percent reduction of greenhouse gas emissions, compared to baseline			
Total	0 *(0)*	26.0 *(43.5)*	19.8 *(63.6)*
Group i1	0 *(0)*	19.6 *(45.8)*	17.5 *(61.1)*
Group i2	0 *(0)*	23.9 *(45.4)*	21.1 *(60.2)*
Group i3	0 *(0)*	34.2 *(39.3)*	20.8 *(70.7)*
Mean (*SD*) weekly video streaming duration in hours			
Total	23.0 *(12.9)*	17.1 *(9.8)*	17.1 *(12.3)*
Group i1	24.8 *(11.0)*	20.3 *(10.2)*	21.1 *(14.0)*
Group i2	23.4 *(15.6)*	15.0 *(8.2)*	15.1 *(10.4)*
Group i3	21.0 *(12.2)*	15.9 *(10.3)*	15.0 *(11.3)*
Mean (*SD*) percentage of high resolution settings			
Total	76.7 *(30.5)*	69.4 *(34.0)*	67.1 *(38.1)*
Group i1	80.0 *(29.8)*	77.6 *(33.8)*	74.3 *(36.3)*
Group i2	76.5 *(29.5)*	70.0 *(29.6)*	66.5 *(37.2)*
Group i3	73.8 *(32.5)*	61.0 *(36.8)*	60.5 *(40.4)*

^a^i1 = information only, *n* = 31

^b^i2 = intervention + goal setting, *n* = 29

^c^i3 = intervention + goal setting + feedback,* n* = 32

### Main Analysis

We obtained a significant main effect of time, *F*(2, 178) = 28.735, *p* < 0.001, partial η^2^ = 0.24. Compared to t1, there was a significant reduction of GHG emissions at both t2 (mean difference = 714 grams CO_2_e, *p* < 0.001) and t3 (mean difference = 623 grams CO_2_e, *p* < 0.001). There was no significant difference between t2 and t3. This confirms Hypothesis 1, according to which all three interventions have an effect on GHG emissions. There was also a significant main effect of group, *F*(2, 89) = 4.472, *p* = 0.014, partial η^2^ = 0.09. Bonferroni-corrected post hoc comparisons revealed that the GHG emissions were significantly lower in the i3 than in the i1 group (mean difference = 706 grams CO_2_e, *p* = 0.013). However, there was no significant interaction, *F*(4, 178) = 0.475, *p* = 0.75, implying that the intervention did not account for these group differences. Therefore, Hypotheses 2 and 3 are not supported.

We ran an additional analysis on the relative carbon reduction per participant at t3 (mean = 0.26, *SD* = 0.43) and t2 (mean = 0.20, *SD* = 0.64), both compared to t1. A mixed 3*2 ANOVA with group as the between- and time (t2 vs. t1; t3 vs. t1) as the within-participant factor revealed no main effect of group (*F* < 1), indicating that relative changes to GHG emissions do not yield different results than absolute changes. To control for seasonal effects, we calculated an ANOVA with wave (January–March 2021 vs. June–August 2021) as the between- and time (t1 vs. t2 vs. t3) as the within-participant factor. The main effect of wave was not significant (*F* < 1), nor was the interaction between wave and time, *F*(2, 180) = 1.874, *p* = 0.17, indicating that the intervention effect is not confounded with seasonal differences.

### Exploratory Analyses

We explored whether the participants in the i2 and i3 groups (*n* = 61) had attained their 20% reduction goal after week 4 and week 7. The majority had attained the reduction goal in week 4 (68.9%) and maintained it in week 7 (63.9%). There was no significant difference between these two groups regarding goal attainment (χ^2^ < 1).

We further investigated the types of behaviour the participants exerted to reduce the carbon impact of their video-streaming activities. First, we looked at streaming duration. A 3*3 mixed ANOVA yielded a significant main effect of time, *F*(2, 178) = 22.290, *p* < 0.001, partial η^2^ = 0.20. Compared to t1, participants reduced their streaming time by approximately six hours per week at t2 and t3 (*p*s < 0.001; see Table 3). There was no difference between groups, *F*(2, 89) = 2.076, *p* = 0.132, and no interaction, *F*(4, 178) = 1.068, *p* = 0.37. This indicates that the participants followed a sufficiency strategy, regardless of the intervention type.

Second, we examined the extent to which the participants employed the two efficiency strategies suggested by Suski et al. (2020): device choice and resolution setting. Regarding device choice, we analysed whether the proportion of streaming time using the most efficient device (i.e., the smartphone) increased and whether the proportion of streaming time using the most inefficient device (i.e., the smart TV) decreased following the intervention. We conducted 3*3 mixed ANOVAs with the participants using the smartphone (*n* = 90) and the smart TV (*n* = 71) at least once during the measurement period, respectively. There was no significant main or interaction effect in either analysis.

For resolution, we split the reported settings into a lower category which comprises exact settings of 480 pixels or lower and the verbalized options “low quality” and “medium quality,” and a higher category including the exact settings of 720 pixels or higher and the verbalized options “high quality” and “ultra HD.” Using the same ANOVA design as in the previous analyses, we examined how the relative frequency of higher resolution settings across devices, platform types, and days per week changed during the intervention, without taking into account the streaming durations per setting. The main effect of time was significant, *F*(2, 178) = 7.670, *p* = 0.001, partial η^2^ = 0.08. Compared to t1, the proportion of higher resolution settings decreased by 7.2% at t2 (*p* = 0.004) and 9.6% at t3 (*p* < 0.009; Table 3). There was no significant main effect of group, *F*(2, 89) = 1.203, *p* = 0.30, and no significant interaction (*F* < 1). Therefore, it appears that participants of all intervention groups lowered their resolution settings, thus applying an efficiency strategy.

Finally, we analysed how tracking tool use is associated with streaming duration, resolution settings, and GHG emissions. Given that the majority of participants indicated never (40.2%) or always (30.4%) using a tracking tool, we decided to split them into a group of “users,” who used a tracking tool at least sometimes (*n* = 48) and “non-users,” who indicated rarely or never using a tracking tool (*n* = 44). Three mixed 2*3 ANOVAs were conducted, with tracking tool use (non-user vs. user) and time as predictors and streaming duration, resolution setting frequencies, and GHG emissions as criteria, respectively. The main effects of tracking tool use and interactions were not significant in any of these analyses.

## Discussion

In the present experiment, we investigated the effects of information only (i1), information with goal setting (i2), and additional feedback (i3) on the reduction of the carbon impact associated with video streaming. Compared to the baseline (week 1), the GHG emissions were significantly reduced in the course of the intervention (weeks 2–4) and during the follow-up measurement period (week 7) in all experimental conditions, confirming our basic assumption that informational strategies of behaviour change (Nemati & Penn, 2020), in combination with self-monitoring, work in the context of video streaming. In contrast to our expectations, goal setting and feedback did not yield additional benefits to information provision. Exploratory analyses showed that the participants in all groups reduced the streaming duration and lowered the resolution settings owing to the intervention, while the intervention did not predict changes in the device choice. Goal attainment appeared not to be affected by feedback.

### Knowledge-Based Intervention

The present data provide evidence that information can bring about a considerable pro-environmental behaviour change, resulting in a mean reduction of more than 19% of CO_2_e during the intervention and 17% in the follow-up period in the i1 condition (Table 3). This finding is not in line with earlier notions of a knowledge-action gap which indicated that environmental knowledge is a necessary but not a sufficient predictor of pro-environmental behaviour (e.g., Kollmuss & Agyeman, 2002). We identify two reasons that a knowledge-based intervention appears to be sufficient in the present case. First, digital activities such as video streaming have, to date, been paid little attention by campaigns promoting pro-environmental behaviour (Suski et al., 2020), indicating that there is a need to raise awareness of the consequences of such activities for the climate. Under the caveat that the GHG reduction in the i1 group cannot be attributed to information alone but to information combined with self-monitoring, our finding that information contributes significantly to behaviour change in under-investigated fields of action provides a promising path that needs further investigation.

Second, the knowledge-based intervention in our experiment provides both system- and action-related information. In past research on energy-saving behaviours, such a combination of different information types proved to be more effective than one information type alone (Dogan et al., 2014; Osbaldiston & Schott, 2012). This can be explained theoretically by stage models of behaviour change (Bamberg, 2013; Pelletier & Sharp, 2008), according to which system-related information increases problem awareness, while action-related information supports the shift from problem- to solution-oriented thinking and indicates the feasibility of subsequent action. By this means, people can progress to advanced stages of behaviour change that directly precede action.

### Goal Setting and Goal Attainment

The majority of participants who were assigned a goal to reduce the carbon impact of their streaming activities by 20% achieved this goal directly after the intervention and maintained it three weeks later. The average reduction was 24% after the intervention and 21% in the follow-up period for the i2 group, and 34% after the intervention (21% in the follow-up period) for the i3 group. These reductions are relatively high, compared to similar experiments applying goal-setting strategies to energy-saving behaviours (Abrahamse et al., 2007; Becker, 1978; Karp et al., 2016). Despite this, there is no evidence that goal setting and goal-based feedback added significantly to the carbon reductions brought about by system- and action-related information. One possible explanation is that the self-monitoring effect of the diary method in all three conditions overlaps the effects that goal setting and feedback may have exerted in addition to information. By tracking their own streaming behaviour, which was required for the diary method, participants were provided with self-feedback that pertained to the three behavioural strategies on which the GHG emissions depend (streaming duration, device choice, and resolution setting). Participants who did not receive CO_2_-based feedback may have used this behavioural feedback as a proxy and adapted their behaviour accordingly. Based on this, they could have conceived their own behavioural goals and adapted them in the course of the intervention, even in the i1 condition. Therefore, a valid conclusion could be that *impact-based* goals and feedback do not add to the effect of pre-existing behavioural goals and behavioural self-feedback.

Alternatively, the provided information may have already exerted such a large impact on video streaming behaviour that there was virtually no further reduction potential left for additional goal setting or feedback. It could be that the participants exhaustively used two different strategies (i.e., reduced their streaming time and lowered the resolution settings) only after having received the information. Further carbon reductions may have also required changes to the device choice (Suski et al., 2020), which was perhaps not feasible or acceptable for the participants within the seven-week study period. The 20% reduction goal may also not have been ambitious enough for the specific behaviour targeted in this study. Earlier studies such as those by Becker (1978) and Abrahamse et al. (2007) addressed household energy use in a more comprehensive manner, involving a bundle of diverging behaviours; we referred to Becker (1978) for goal selection due to the lack of previous work specifically addressing the eco-friendly use of digital devices. However, the behaviour of interest in our study was restricted to video streaming as a leisure activity, which is one of many divergent activities in the digital world (albeit the most carbon-intensive one). The participants may have switched to similar activities, such as gaming or watching regular TV, that were not considered in this study in terms of their climate impact. Moreover, increasing the efficiency of streaming by lowering the resolution or co-viewing with others on the same screen could be an easy way to substantially reduce the individual carbon impact of streaming. The mean relative reduction of GHG emissions in the information-only group already approached the 20% reduction goal of the other two groups, thereby supporting the reasoning that this goal may not have been as ambitious as initially thought. A study that varied the difficulty of goal achievement systematically (Reese & Junge, 2017) suggested that meeting a behavioural challenge is significantly associated with efficacy beliefs if the goal is neither too easy nor too difficult. Given that feedback operates mainly through efficacy beliefs (Kluger & DeNisi, 1996; Schwarzer, 2008), it can be argued that feedback will not have an effect on goal achievement if the goal lacks difficulty. Therefore, future research in this domain should assign more ambitious goals either by increasing the share of GHG emissions to be reduced (e.g., 30% instead of 20%) or extending the area of target behaviour by including video gaming, for instance. Self-assigned and adaptive goals may also be of interest in this context.

### Limitations and Further Directions

The internal validity of our findings is challenged by a combination of two methodological issues, namely the lack of a no-intervention control group and the previously mentioned self-monitoring effect of the streaming diary. This makes it impossible to quantify the effect of information as a single interventional element. It remains for future studies to develop an alternative measurement that does not require the participants to document their streaming activities continuously. Regarding the lack of a no-intervention control, we feared a high dropout rate among participants who would have had to fill in streaming diaries without knowing why they were doing so. As an alternative, such a control group could have received information framed economically instead of ecologically. However, we cannot be sure if this would really work for a no-intervention control because there is no study, to our knowledge, that has applied information framed economically to this specific type of behaviour. The results of economic information applied to a broader range of energy-saving behaviours are quite heterogeneous, ranging from positive outcomes (e.g., Brandsma & Blasch, 2019) to backfire effects (Schwartz et al., 2015). Adding a non-normative information condition to the experimental design used here would therefore require a more sophisticated design that also involves, for example, an egoistic goal framing (Lindenberg & Steg, 2007) and corresponding feedback.

Another limitation that may affect our sample stems from its being made up largely of university students and hence being biased towards younger age, female gender, and higher education. This limits the generalisability of our findings because these sample characteristics tend to be associated positively with ecological awareness and pro-environmental behaviour (e.g., Whitley et al., 2018). On the one hand, the effectiveness of information provision could be overestimated because people with high ecological awareness are particularly susceptible to ecological information. On the other, this would mean that even students with comparably high ecological awareness were not aware of the ecological consequences of video streaming before participating in this study, which confirms the notion that online behaviours such as video streaming are underrepresented in ecological campaigns. One good reason to choose a younger sample is that young adults use online platforms particularly often (Beisch & Schäfer, 2020); addressing this group should hence be particularly effective in terms of energy and GHG savings. Future research in this area should recruit a larger and more diverse sample that could reveal more generalizable results and enable additional analyses of sample characteristics, such as age or educational level.

A further critical point is that this study is indecisive about psychological predictors of behaviour change, as they were not included in the survey. We decided not to include such predictors because this would have placed further demands on the participants and may have caused further testing effects in the long-term study. Moreover, the processes anticipated in models of pro-environmental behaviour change diverge substantially regarding their rationale. According to the stage model of self-regulated behaviour change (Bamberg, 2013), people start acting pro-environmentally after having developed a personal norm (i.e., feeling morally obliged to do so; van der Werff & Steg, 2015). Pelletier and Sharp’s (2008) model provides that pro-environmental behaviour change follows a self-determined motivation to do so, which draws on the satisfaction of autonomy, competence, and social relatedness needs (Ryan & Deci, 2000). Therefore, addressing the specific motivations behind ecologically sustainable online behaviours could be a promising field of research. This may particularly pertain to the social dimension of these behaviours, in two ways. First, online behaviours are largely motivated by social relatedness needs (Cole et al., 2017); in the case of video streaming, these needs can be sustainably satisfied by co-viewing, for example. Second, the carbon reductions of more sustainable streaming (in this study, 700 grams CO_2_e per person after the intervention and 600 grams CO_2_e per person in the follow-up week) are negligible, unless they are carried out by many. Therefore, interventions conceiving the GHG impact of online behaviours as a collective challenge and promoting group-based efficacy beliefs (e.g., Hamann & Reese, 2020; Jugert et al., 2016; Reese & Junge, 2017) could be a useful direction for future interventions.

Our results give a first insight into the application of informational interventions aiming to reduce the carbon impact of digital behaviours. The main finding is that providing system- and action-related information in combination with self-monitoring appears to be sufficient, probably because the general public has limited awareness of the ecological impact of these types of behaviour and options to reduce this impact. If a joint initiative of policy makers, streaming providers, and engaged users applied such an intervention to a greater part of the population, this would probably result in a considerable reduction of GHG emissions. However, such user-centred interventions cannot replace infrastructural changes: Server farms need to be repowered based on renewable energies, and end-user devices need to be fairly produced, long-living, repairable, and recyclable. To do justice to this, life cycle analyses (e.g., Suski et al., 2020) should be used to investigate sustainable digital behaviours, especially when acquisition behaviours, such as device purchase or platform choice, are addressed.

## Data Availability

The datasets generated during the current study are available in the Open Science Framework repository, https://osf.io/kazy7/.
